# Relaxation and Amorphous Structure of Polymers Containing Rigid Fumarate Segments

**DOI:** 10.3390/polym14224876

**Published:** 2022-11-12

**Authors:** Yasuhito Suzuki, Takahito Kano, Tsuyoshi Tomii, Nagisa Tsuji, Akikazu Matsumoto

**Affiliations:** 1 Department of Applied Chemistry, Graduate School of Engineering, Osaka Prefecture University, 1-1 Gakuen-cho, Naka-ku, Sakai 599-8531, Osaka, Japan; 2 Department of Applied Chemistry, Graduate School of Engineering, Osaka Metropolitan University, 1-1 Gakuen-cho, Naka-ku, Sakai 599-8531, Osaka, Japan

**Keywords:** *β* relaxation, poly(fumarate)s, amorphous structure, differential scanning calorimetry

## Abstract

The physical properties of polymers are significantly affected by relaxation processes. Recently, we reported that poly(diethyl fumarate) (PDEF) shows two thermal anomalies on DSC measurement, despite the fact that it is a homopolymer. We attribute these two relaxations *α* relaxation and *β* relaxation, respectively. In this study, we investigate the two relaxations of fumarate-containing polymers by DSC, solid-state NMR, and X-ray scattering. The two relaxations are present even in a copolymer of diethyl fumarate and ethyl acrylate with fumarate segments of 30%. We used poly(methyl methacrylate) (PMMA) as a model polymer for comparison, since there are detailed investigations of its dynamics and physical properties. Solid-state NMR indicates that the very local relaxation of poly(fumarate)s is not significantly different from that of PMMA. The tensile test showed that PDEF is still brittle at above *β* relaxation temperature and below *α* relaxation temperature. It was revealed that a structural anisotropy appeared when PDEF was extended at around *α* relaxation temperature. We discuss the effect of the glassy packing of the rigid polymer chain including the DEF segments on the strong *β* relaxation behavior. Our data provide insight into the microscopic mechanism of *β* relaxation of vinyl polymers.

## 1. Introduction

At glass transition, the physical properties of amorphous polymer significantly change [1,2]. Glass transition is governed by segmental or *α* relaxation. As it is also called liquid-to-glass temperature, a glassy solid polymer softens to polymer melt or rubbery polymer depending on its molecular weight upon heating. The elastic modulus of glassy polymers is typically 2 to 4 GPa [3]. Interestingly, this value is universal with different kinds of polymers. In contrast, the strength of polymers hugely varies depending on their chemical structure and amorphous packing [4]. Dynamic mechanical analysis (DMA) measures the mechanical response to a sinusoidal mechanical perturbation as a function of temperature at a fixed frequency [5]. This spectroscopy depicts *α* relaxation as a sudden decrease in the real part of the modulus as well as the peak of the imaginary part of the modulus. Dielectric spectroscopy captures the frequency and temperature dependence of the complex dielectric permittivity. It enables the measurement of the characteristic time of the relaxation processes in a broad frequency range (i.e., 10^−2^ Hz to 10^7^ Hz) [6]. The most commonly used method is differential scanning calorimetry (DSC) [7,8,9]. It captures glass transition as a step, reflecting the change in heat capacity.

In general, from a homopolymer, only a single thermal anomaly is observed in the DSC curve. Although there are multiple relaxations in amorphous polymers (i.e., *β* and γ relaxations), their intensity is so tiny that normal DSC cannot detect them [10,11]. When more than two different components are present in polymer alloys or in copolymers, multiple anomalies can be observed [12]. However, still, each glass transitions correspond to different chemical compositions. A few reported exceptions are polymers with long side chains [13,14,15]. They possess two thermal anomalies in DSC. They are attributed to two *α* relaxations. One is from the glass transition of the main chain, and the other is from the independent glass transition of the side chain. Classically, *β* relaxation of polymers is considered as the local side-chain motion [5]. The universality of *β* relaxation has been shown because *β* relaxation is discovered from rigid molecules without side chains [16]. Some *β* relaxation is regarded as a precursor of *α* relaxation [17]. Recently, *β* relaxation has drawn more attention because it may govern diverse properties related to transportation [18,19], crystallization kinetics [20,21], and mechanical properties [22,23,24,25]. Some report suggests that the intensity of the *β* relaxation is intimately related to the amorphous packing [26]. Recently, we reported two thermal anomalies on DSC from poly(diethyl fumarate) (PDEF) [27]. The side chain of PDEF is an ethyl group. It is significantly shorter than the previously reported homopolymers possessing two glass transitions. Based on the DMA and dielectric spectroscopy, we concluded that the two thermal anomalies of PDEF correspond to *α* relaxation and *β* relaxation, respectively [26,27,28,29]. 

Fumarates are categorized as 1,2-disubstituted ethylene. Generally, it is difficult to obtain poly(substituted ethylene) with reasonable molecular weight because of the extremely low propagation kinetics of the monomer [30,31,32]. Exceptionally, poly(fumarate)s can be obtained. It is because the slow termination rate balances with the slow propagation rate [33]. Considering the structure of poly(fumarate)s, it does not possess a methylene spacer (-CH_2_-) in the main chain. In other words, the rotational barrier along the main chain is huge [34]. Other than radical polymerization of fumarates, poly(substituted ethylene) can also be prepared by C1 polymerization [35,36,37,38,39]. Metal catalyst enables the polymerization of one carbon atom in the main chain at once. Our previous studies revealed unusual physical properties of poly(fumarate)s [17,18,40,41]. Especially the intensity of the *β* relaxation is strong. Although the knowledge of the detailed motion of the *β* relaxation is limited, the *β* relaxation of poly(methyl methacrylate) [42] and poly(ethyl methacrylate) [43] is the rotational motion along the main chain according to the 2D solid-state NMR. The strong *β* relaxation of poly(fumarate)s seems to be linked to the rotational barrier along the main chain. 

The aim of this study is to further investigate the physical properties of poly(fumarate)s. Thermal properties are investigated using copolymers containing different fumarate fractions. The local dynamics are captured by solid-state NMR. The tensile test is conducted at different temperatures. The amorphous structures after the extension were investigated by wide-angle X-ray scattering (WAXS). We discuss the relaxation behavior of poly(fumarate)s in conjunction with the amorphous packing.

## 2. Materials and Methods

### 2.1. Materials

**Scheme 1:**
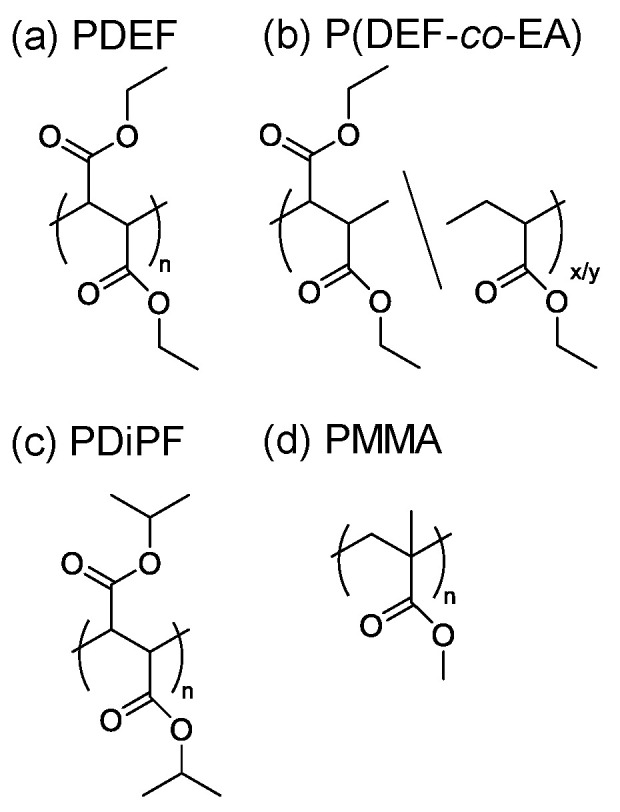
The chemical structures of polymers investigated in this study. (**a**) PDEF, (**b**) P(DEF-*co*-EA), (**c**) PDiPF, and (**d**) PMMA.

Diethyl fumarate (DEF), diisopropyl fumarate (DiPF), ethyl acrylate (EA), and dimethyl 2,2′-azobis(2-methylpropionate) (MAIB) are purchased from Fujifilm Wako Pure Chemical Corporation, Tokyo, Japan. Monomers (DEF, DiPF, and EA) were used without further purification. The initiator (MAIB) was purified via recrystallization in hexane. PDEF, poly(diisopropyl fumarate) (PDiPF), and P(DEF-*co*-EA) were synthesized via radical polymerization as described elsewhere [40]. It is briefly explained below. The remaining oxygen was removed from the monomer and initiator in a flask via freeze–thaw cycles three times. Under the nitrogen atmosphere, the reaction was conducted at 80 °C for three hours. After stopping the reaction by freezing using liquid nitrogen, the polymer was dissolved in a small amount of chloroform. It was precipitated in hexane/ethyl acetate = 30/1 (*v*/*v*). The polymer was dried at 90 °C under a vacuum for 5 h. The molecular weight and composition of the copolymer are summarized in Table 1. PMMA was purchased from Sigma-Aldrich. The number average molar mass (*M*_n_) and weight average molar mass (*M*_w_) are 15,500 g/mol and 25,600 g/mol, respectively. PDEF (*M*_n_ = 15,500, *M*_w_ = 25,600) synthesized in our previous research [16] was used for the data presented in Scheme 1.

### 2.2. Differential Scanning Calorimetry (DSC)

The thermal anomalies were detected using DSC 250 from TA Instruments (New Castle, DE, USA). DSC was calibrated using indium as a reference. Typically, 3 mg of the sample was placed in a hermetic DSC pan. The data were obtained from the modulated DSC. The heating speed, the modulation amplitude, and the modulation period were 3 °C/min, 2 °C, and 60 s, respectively.

### 2.3. Nuclear Magnetic Resonance Spectroscopy (NMR)

JEOL ECX-400 was used for NMR measurements. The fraction of fumarate segments in the synthesized copolymer was determined by ^1^H NMR spectroscopy. The sample was dissolved in deuterated chloroform. The NMR spectra were recorded from −1 to 9 ppm of the chemical shift (δ). The peak from tetramethylsilane was used as δ = 0.

^13^C CP/MAS solid-state NMR measurement was conducted to obtain the spin-lattice relaxation time ($T_{1}^{c}$) from 20 °C to 80 °C. The Torchia method was used [44]. The sequence was pre-installed in the Delta software from JEOL (torchiat1_cpmas). The sample was placed in a sample rotor made of ceramic. The magic angle spinning frequency was 4 kHz, and the contact time was 2 ms. 

### 2.4. Size Exclusion Chromatography (SEC)

The molecular weight distribution was analyzed using SEC with the Tosoh TSK-gel GMHHR-N as a column at 40 °C in a chamber (Chromato Science CS-300 C). The polymer was detected by RI detector (JASCORI-2031-Plus). The flow rate was 0.8 mL/min. The molecular weight was calibrated with polystyrene standard from *M*_n_ = 2890 to *M*_n_ = 96,500.

### 2.5. Dynamic Mechanical Analysis (DMA)

DMA was conducted using DMA 6100 (Seiko Instruments Inc., Tokyo, Japan). Sinusoidal strains with an amplitude of 10 μm at 1 Hz were applied with a dual cantilever mode. The heating speed was 2 °C/min. A rectangular sample (5 × 1 cm) was prepared using a mold. The mold was placed in a hot press, AH-2003 (AS ONE CORPORATION, Osaka, Japan). The hot press was heated to 180 °C. Approximately 1 MPa was applied for 1 h. The mold was taken out from the hot press and cooled down in the air. 

### 2.6. Tensile Test

Autograph AGSX 5 kN (Shimadzu Corporation, Ltd., Kyoto, Japan) was used for the tensile test. The temperature was controlled in a thermostatic chamber TCR1A-200T. A rectangular sample (5 × 1 cm) was prepared by the same method as the DMA sample. The tension rate was 1 mm/min. The engineering modulus was obtained from the initial slope of the stress–strain curve, in which the strain is from 0.05% to 0.25%.

### 2.7. Wide-Angle X-ray Scattering

X-ray scattering measurements were performed at BL40B2 beamline of SPring-8 (Japan Synchrotron Radiation Research Institute, Hyogo, Japan). The specimen prepared for the tensile test was fixed in the sample folder using Kapton tape. PILATUS3 S 2M was used as a detector. The distance was 327.4 mm. The wavelength (λ) of the synchrotron beam was 1.00 Å. The exposure time was 4 s.

## 3. Results and Discussions

**Figure 1:**
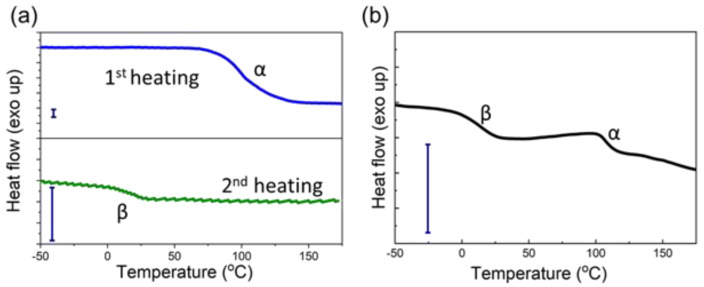
DSC profiles of PDEF with different thermal histories. (**a**) The sample was quenched from 180 °C. While mainly *α* relaxation is observed in the first heating, *β* relaxation was exclusively observed in the second heating. (**b**) The sample was quenched from 150 °C. Two thermal anomalies corresponding to *α* relaxation and *β* relaxation can be seen.

The chemical structures of the polymers used in this study are summarized in Scheme 1. The list of polymers synthesized, and their molecular weights are summarized in Table 1. As discussed in the introduction, the absence of the methylene spacer in the main chain is the key to the characteristic features of poly(fumarate)s, including PDEF and PDiPF. The chemical structures of side chains in PDEF and poly(ethyl acrylate) (PEA) are the same. Therefore, random copolymerization of DEF and EA enables systematic change in the concentration of methylene spacers. Figure 1a presents DSC data obtained from PDEF quenched from 180 °C. It is well known that the DSC curve of the amorphous polymer depends on thermal history. Reflecting the different packing of the amorphous structure, it often shows slightly different glass transition temperatures. In the case of PDEF, the behavior is fundamentally different from normal amorphous polymer because of the unusually strong *β* relaxation. In the first heating, a single step at 101 °C corresponding to *α* relaxation is observed. On the other hand, in the subsequent second heating, *β* relaxation at 18 °C is exclusively detected. Figure 1b shows DSC profiles of PDEF quenched from 150 °C. It shows two thermal anomalies at 14 °C and at 106 °C. This behavior is very unusual as a homopolymer and has been discussed in detail in our previous paper [16]. It should be stressed that all the DSC profiles in Figure 1 are obtained from the same sample but with different thermal histories. As we pointed out in the previous paper [16], the intensities of two relaxation processes sensitively depend on the thermal history.

**Figure 2:**
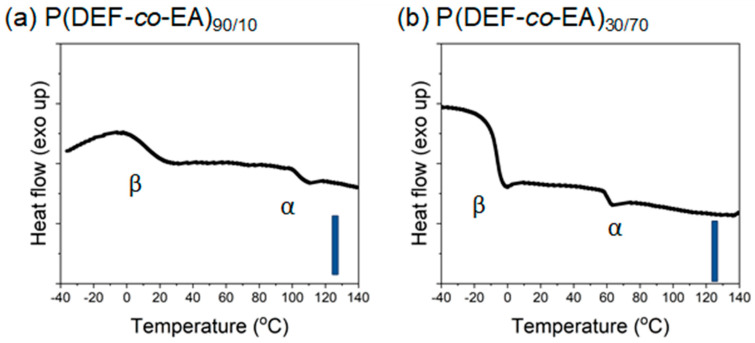
DSC profiles of P(DEF-*co*-EA)_90/10_ and P(DEF-*co*-EA)_30/70_. There are two thermal anomalies even in the copolymer containing the 30 % DEF segment. The scale bar corresponds to 0.05 W/g. The other DSC data of copolymers with different fumarate segments are provided in the supporting information. The sample was quenched from 150 °C.

In Figure 2, the copolymers of DEF and EA with different fumarate segments are analyzed. The data are obtained from the first heating with a scanning speed of 3 °C/min. The data corresponding to other copolymers are provided in Figure S1. Some of the data clearly shows two thermal anomalies. Even when the DEF content is as low as 30%, it shows two clear steps. This means that the characteristic feature of PDEF (i.e., strong *β* relaxation) is present with the fumarate fraction of 30%. The physical properties of PDEF and PEA are fully different. This can be seen by the *T*_g_ presented in Table 2. The *T*_g_ of PEA is at −23 °C. It is clear from the literature that PEA shows only one thermal anomaly on DSC measurements [45]. As the EA content increases in P(DEF-*co*-EA), *α* relaxation temperature decreases steeply while *β* relaxation shifts slightly. Here, terminology should be carefully used. As it has been pointed out, the terminology of especially *β* relaxation is confusing because in some cases, it is used based only on the fact that the relaxation is secondary, while it is named based on the molecular mechanism in other cases [20]. We refer two relaxation processes in PDEF to *α* and *β* relaxations, respectively. It is reasonable to assume that the two relaxations captured in P(DEF-*co*-EA) would have a similar relaxation mechanism to that of the homopolymer of PDEF.

**Figure 3:**
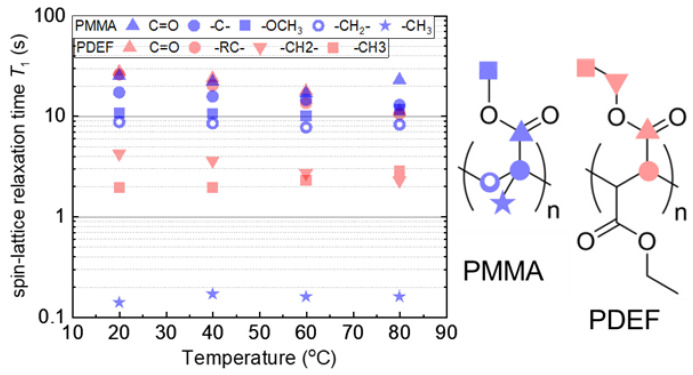
Temperature dependence of spin-lattice relaxation time *T*_1_ for PMMA (blue symbol) and PDEF (red symbol). The symbols on the chemical structure correspond to *T*_1_ plotted on the graph.

We applied solid-state NMR to investigate the local dynamics. The previous study has applied solid-state NMR to PDiPF and poly(dicyclohexyl fumarate) (PDCHF) [46,47]. Since our recent results suggest unusually strong *β* relaxation of poly(fumarate)s, it is of interest to investigate the $T_{1}^{c}$ dependence on temperature, especially around *β* relaxation. Figure S2 presents the $T_{1}^{c}$ of PDiPF from 20 °C to 80 °C. The data are in good agreement with the reported data [45]. Figure 3 depicts $T_{1}^{c}$ of PDEF. The data are very similar to that of PDiPF in this temperature range. Except for CH_3_ carbon on the side chain, the value of $T_{1}^{c}$ decreases as the temperature increases, indicating that these carbons are in the slow-motion region. On the other hand, CH_3_ carbon belongs to the fast-motion region. In the same temperature range, $T_{1}^{c}$ of PMMA is obtained. Consistent with previous research [48], no significant change in $T_{1}^{c}$ of PMMA is observed in this temperature range. Interestingly, both PDEF and PMMA have similar $T_{1}^{c}$ value of carbonyl carbon. Although there is a small discrepancy at 80 °C, the $T_{1}^{c}$ value of PMMA is in between that of PDiPF and PDEF. In other words, the effect of the rotation barrier on the carbonyl carbon is not observed in this temperature range. It is noted that the relation between spin-lattice relaxation time and correlation times ($\tau_{0}$) characterizing molecular rumbling is not straightforward. In fact, the spin-lattice relaxation time is a concave down parabolic function against $\tau_{0}$ [49]. At least from the temperature range we investigated, no clear sign of *β* relaxation was observed. It is because the timescale of the $T_{1}^{c}$ relaxation is much faster than the time scale of DSC measurements with a scanning speed of 3 °C/min. We concluded that the very local dynamics of poly(fumarate)s are not significantly different from that of PMMA.

**Figure 4:**
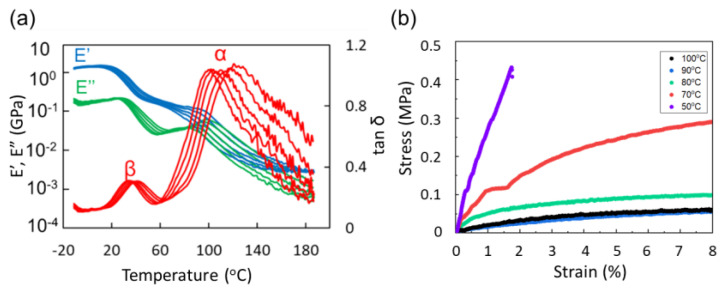
(**a**) DMA profile of PDEF as a function of temperature at five different frequencies, including 0.1 Hz, 0.5 Hz, 1 Hz, 2 Hz, and 5 Hz. (**b**) Stress–strain curve of PDEF at various temperatures indicated in the inset.

Figure 4a shows the DMA data of PDEF. Both *α* and *β* relaxation are clearly seen. The main difference between poly(fumarate)s and other (meth)acrylic polymers is the strong intensity of the *β* relaxation. The storage modulus of PDEF drops from ca. 2 GPa to ca. 0.2 GPa. In order to better understand the molecular motion corresponding to these relaxation processes, a tensile test was conducted. Figure 4b presents stress–strain curves of PDEF at different temperatures. The engineering modulus is summarized in Table 3. These values are in the same order as the storage modulus obtained from DMA at each temperature. For example, the modulus of PDEF at 50 °C (i.e., 410 MPa) is comparable to the storage modulus obtained from DMA presented in Figure 4a. The important finding is that the polymer is still brittle at 50 °C, which is above *β* relaxation temperature. Although the relaxation temperature depends on frequency (i.e., characteristic time), it can be said that 50 °C is below *α* relaxation temperature in the time scale of the tensile test. The stress–strain curve is almost linear till the break, indicating an elastic response. Above 70 °C, plastic deformation is observed. Considering the DMA curve presented in Figure 4a, it is suggested that the molecular motion related to *α* relaxation process is partially active at 70 °C. The tensile test data suggest that the *β* relaxation is a local motion that does not lead to the flow of the polymer. On the other hand, the molecular motion associated with *α* relaxation causes plastic deformation.

**Figure 5:**
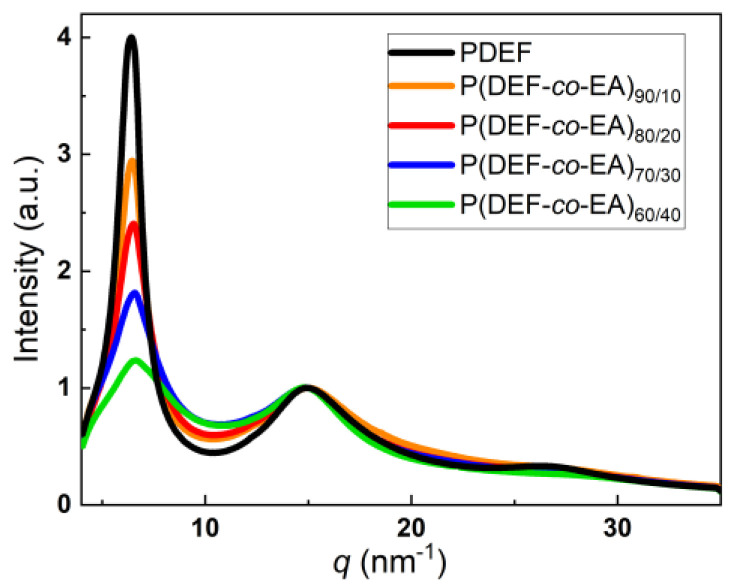
WAXS profiles of P(DEF-*co*-EA) with different fumarate fractions. The intensity is normalized at the peak position at *q* = 15 nm^−1^.

WAXS measurements of amorphous polymer show a so-called amorphous halo, which reflects the amorphous packing [50,51,52]. In the previous study, we pointed out that the amorphous halo of PDEF is unique in comparison to that of PMMA. PDEF shows a strong peak at low *q* at 6.5 nm^−1^ [18]. Figure 5 depicts WAXS profiles of P(DEF-*co*-EA) with different fumarate fractions. The peak of the amorphous halo at low *q* is characteristic of poly(fumarate)s. The length scale of the peak is around 9.7 Å. It is comparable to twice the value of the side chain length. It may correspond to the cross-sectional diameter of the polymer, taking the polymer chain as a rigid rod. The intensity of the low *q* peak increases systematically as the DEF segment increases. The *q* value at the maximum intensity is almost constant regardless of the fumarate fractions. In other words, while the enhancement of the rotation along the main chain decreases the maximum intensity of the peak, it does not affect the peak position. It is worth mentioning that the amorphous peak at low *q* is also observed in polymers that have two *α* relaxations [14,15]. 

**Figure 6:**
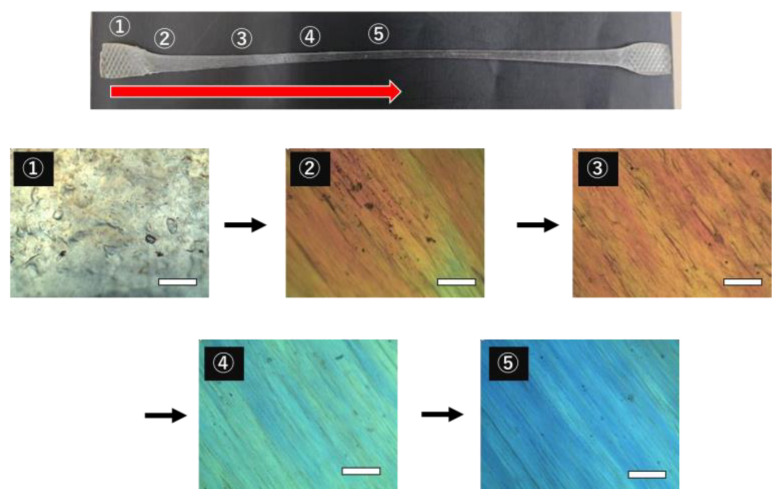
(**Top**) A photograph of a PDEF specimen after a tensile test. (**Bottom**) Polarizing optical images of the specimen at five different locations as pointed out in the photograph. The scale bars are 100 μm.

**Figure 7:**
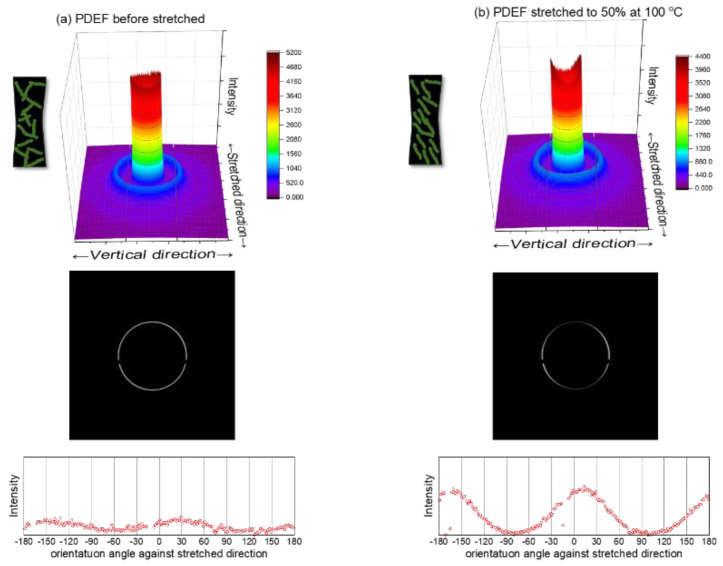
WAXS profiles of PDEF (**a**) before and (**b**) after the 50% extension at 100 °C. (**Top**) Three-dimensional plot, (**middle**) two-dimensional plot, and (**bottom**) the intensity profile in the azimuthal angle are displayed. The intensity is in the arbitral unit.

**Figure 8:**
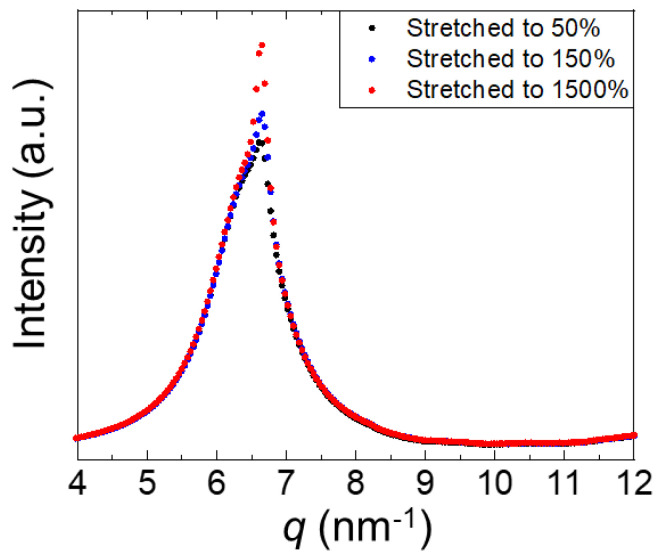
WAXS profile of PDEF after extension at 100 °C for 50%, 150%, and 1500%. The *q* value ranging from 4 to 12 nm^−1^ is presented. The peak of the amorphous halo at *q* = 6.5 nm^−1^ starts to split after extension.

We noticed that there is an indication of local ordering after the tensile test based on polarizing optical microscope images presented in Figure 6. The images are taken at five different positions of the tensile specimen. There is no specific polarization property before the extension. However, after the extension, the polarization properties are different depending on the position of the sample. No clear polarization is observed only at position 1, where there was no extension because it was cramped during the tensile test. Figure 7 shows WAXS profiles before and after the extension of 50% at 100 °C. The data are presented both in 3D and 2D. The intensity profile along the azimuthal angle captures the local orientation. The microscopic ordering of the amorphous structure of PDiPF was mentioned in previous research [34]. This microscopic ordering is only observed from the peak of the amorphous halo at *q* = 6.5 nm^−1^. From the broad second peak of the amorphous halo, there was no indication of the intensity change in the azimuthal angle direction. In addition, we did not find an indication of local orientation when the extension was conducted at 50 °C, which is above the *β* relaxation temperature. Based on this data, the local orientation occurs in conjunction with the molecular motion associated with *α* relaxation and not with *β* relaxation. The one-dimensional plot of intensity as a function of *q* is shown in Figure 8. It can be seen that the peak of the amorphous halo split into two peaks. The sharp peak at *q* = 6.6 nm^−1^ appears in addition to the relatively broad peak at *q* = 6.5 nm^−1^. This implies that *α* relaxation activates the motion of the main chain. Some side chains of the nearby polymer chains are interpenetrated.

Our data support that the two thermal anomalies of PDEF observed in DSC measurement correspond to *β* relaxation and *α* relaxation rather than two *T*_g_s. Therefore, at the temperature range between *β* relaxation and *α* relaxation, PDEF is still brittle and responds elastically upon extension. The strong *β* relaxation is likely to be linked to the amorphous peak at *q* = 6.5 nm^−1^. Due to the rod-like nature of the poly(fumarate)s, it behaves similarly to the main chain liquid crystalline polymers. A possible scenario is as follows. Above *β* relaxation temperature, the side chain cannot fully rotate along the main chain. Hence, the material does not flow upon extension. Nevertheless, the side chain starts to rattle at *β* relaxation. This rattle weakens the amorphous packing. As a result, the storage modulus decreases, and heat capacity changes. The rotation of the side chain along the main chain activates at *α* relaxation. Hence, upon extension, it responds plastically. These dynamics interpenetrate the side chains, resulting in the splitting of the WAXS peak of the amorphous hollow at low *q*.

## 4. Conclusions

The relaxation behavior and amorphous structure of fumarate-containing polymer have been investigated. The rotational barrier of fumarates along the main chain significantly affects the physical properties. In our previous research, we have reported strong *β* relaxation of PDEF. As a result, two thermal anomalies corresponding to *α* and *β* relaxations are detected in DSC, which is very unusual as a homopolymer. In this study, we showed that this characteristic is present in the copolymer of fumarate and acrylate. We confirmed that two thermal anomalies are observed from P(DEF-*co*-EA) containing as low as 30% of fumarate segments. The relaxation behavior obtained by solid-state NMR suggests that the local dynamics of PDEF are not significantly different from that of PMMA. WAXS measurements showed that the amorphous hallow at low *q* is characteristic of the fumarate-containing polymers. After the extension of the PDEF, it showed an anisotropic amorphous structure, indicating an alignment. Our data point out that the glassy packing of PDEF plays an important role in the unusually strong *β* relaxation.

## Figures and Tables

**Table 1:** List of polymers synthesized in this study.

Feed (mol%) DEF/EA	Actual (mol%) ^a^ DEF/EA	*M*_n_	*M*_w_	*M*_w_ */M*_n_
100/0	100/0	13,000	24,000	1.9
90/10	88/12	12,000	24,000	2.0
80/20	80/20	13,000	28,000	2.2
60/40	67/33	17,000	46,000	2.7
30/70	37/63	17,000	39,000	2.3

^a^ Determined by ^1^H-NMR spectra.

**Table 2:** *β* and *α* relaxation temperatures of PDEF, PDiPF, PMMA, PEA, and P(DEF-*co*-EA) with different fractions of fumarate segment. These values are determined by DSC with a scanning speed of 3 °C/min.

	*β* Relaxation Temperature (°C)	*α* Relaxation Temperature (°C)	Ref.
PDEF	27	107	[27]
PDiPF	67	137	[29]
PMMA	-	120	[45]
P(DEF-*co*-EA)_90/10_	15	101	This study
P(DEF-*co*-EA)_80/20_	5	95	This study
P(DEF-*co*-EA)_60/40_	0	75	This study
P(DEF-*co*-EA)_30/70_	−12	60	This study
PEA	-	−23	[45]

**Table 3:** Engineering modulus and ultimate tensile strength of PDEF obtained from tensile test presented in Figure 3b.

Temperature (°C)	Modulus (MPa)	Ultimate Tensile Strength (MPa)
50	410	0.43
70	110	0.36
80	68	0.12
90	14	0.065
100	18	0.065

## Data Availability

Not applicable.

## Supplementary Materials

The following supporting information can be downloaded at: https://www.mdpi.com/article/10.3390/polym14224876/s1, Figure S1: DSC profiles of P(DEF-*co*-EA)_80/20_ and P(DEF-*co*-EA)_30/70_; Figure S2: Temperature dependence of spin-lattice relaxation time *T*_1_ for PDiPF.
