# Supplementary material for: Relaxation and Amorphous Structure of Polymers Containing Rigid Fumarate Segments
Source: Polymers (Basel). 2022 Nov 12;14(22):4876. doi: 10.3390/polym14224876 (PMC9692691; doi:10.3390/polym14224876)
Supplement: Supplementary file 1 [file polymers-14-04876-s001.zip › polymers-2011126-supplementary.pdf]

Supporting Information

Relaxation and amorphous structure of polymers containing rigid fumarate segments

Yasuhito Suzuki[1,2*], Takahito Kano[1], Tsuyoshi Tomii[1], Nagisa Tsuji[1], and Akikazu Matsumoto[1,2*]

1. Department of Applied Chemistry, Graduate School of Engineering, Osaka Prefecture University, 1-1 Gakuen-cho, Naka-ku, Sakai 599-8531, Osaka, Japan

2. Department of Applied Chemistry, Graduate School of Engineering, Osaka Metropolitan University, 1-1 Gakuen-cho, Naka-ku, Sakai 599-8531, Osaka, Japan

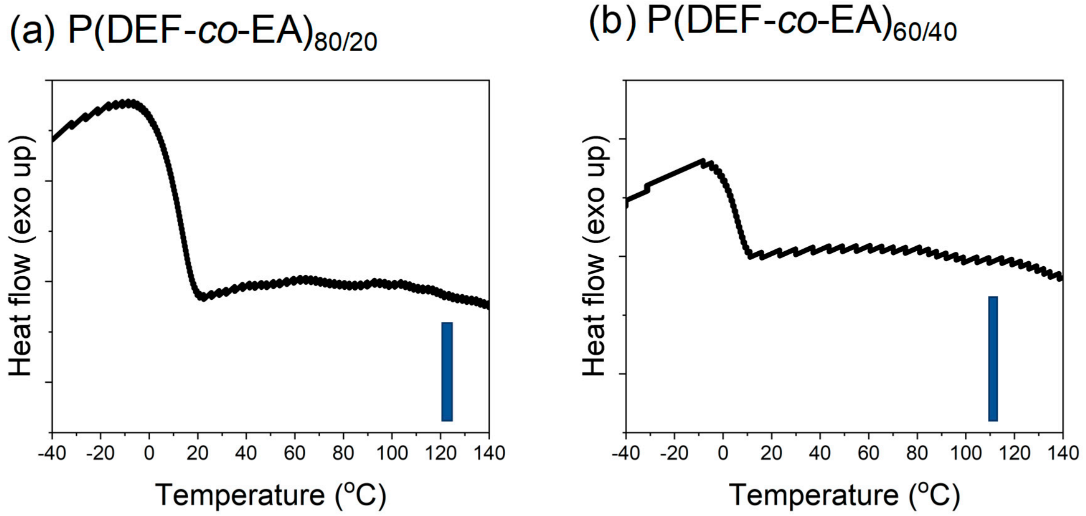

Figure S1. DSC profiles of P(DEF-*co*-EA)$_{80/20}$ and P(DEF-*co*-EA)$_{30/70}$. The scale bar corresponds to 0.02 W/g. The other DSC data of copolymers with different fumarate segments are provided in the supporting information.

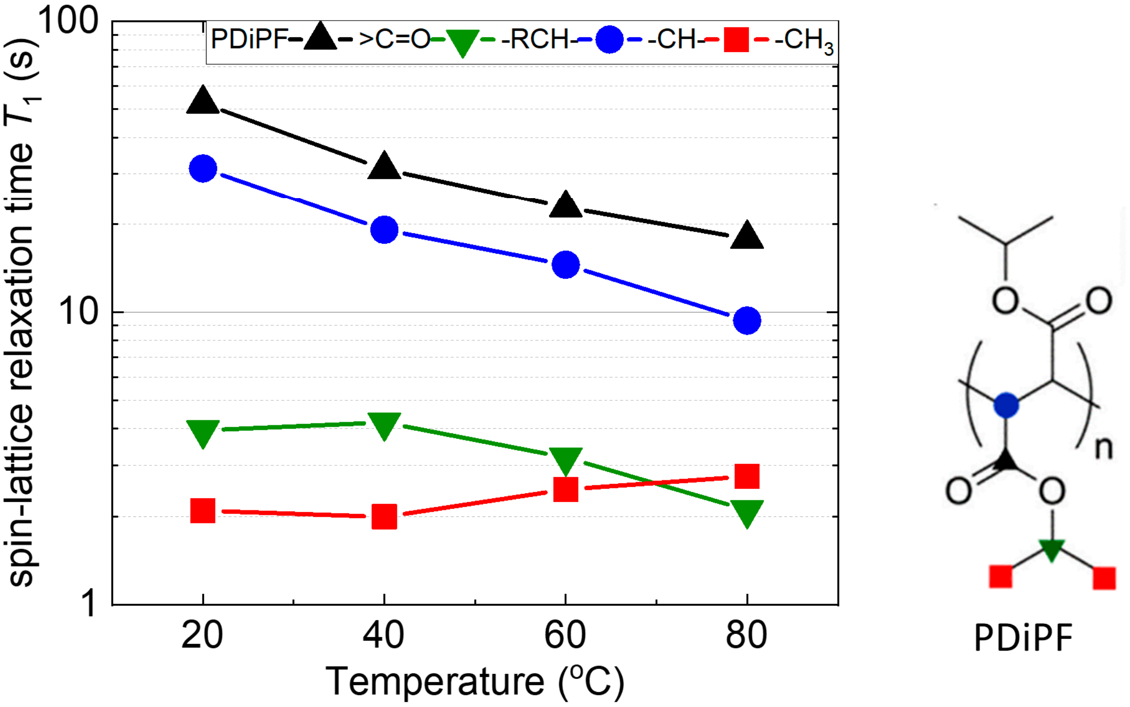

Figure S2. Temperature dependence of spin-lattice relaxation time T_1 for PDiPF.
